# Acute Right Ventricular Failure Triggered by Excessive Fluid Resuscitation in Septic Shock

**DOI:** 10.1155/carm/4817187

**Published:** 2026-06-30

**Authors:** Mohamed Achref Boubaker

**Affiliations:** ^1^ Department of Anesthesiology and Intensive Care Medicine, Bethanien Hospital Moers, Moers, Germany

## Abstract

Acute right ventricular (RV) failure is a serious but underappreciated complication of septic shock resuscitation. Septic shock involves both relative hypovolemia and pathological vasoplegia [1, 2]. Fluid resuscitation is essential early, but excessive volumes can precipitate RV dilation, systolic dysfunction, and venous congestion before the problem is recognized. We report a 68‐year‐old postoperative patient who developed hemodynamic deterioration after 10 L of crystalloid for abdominal sepsis. Bedside echocardiography identified severe RV dilation with a D‐sign, a TAPSE of 10 mm, and a plethoric noncollapsing inferior vena cava (IVC) of 28 mm. De‐resuscitation with diuresis and vasopressors produced transient improvement, but the patient died 1 week later from recurrent sepsis due to an anastomotic leak. The case shows that fluid‐induced RV failure can develop silently and may be reversible if caught early. Earlier echocardiographic monitoring might have allowed earlier recognition before hemodynamic collapse developed.

## 1. Introduction

Right ventricular (RV) failure during septic shock resuscitation may be underrecognized. [1]. Septic shock is a distributive state combining vasoplegia and relative hypovolemia [2]. While early fluid loading is appropriate, fluid responsiveness may diminish rapidly, and continued administration beyond it can precipitate venous congestion and acute RV dilation [3]. Bedside echocardiography allows direct and rapid assessment of RV size, septal configuration, TAPSE, and inferior vena cava (IVC) dynamics. It is recommended in any patient with unexplained hemodynamic instability during sepsis [3].

## 2. Case Report

**FIGURE 1:**
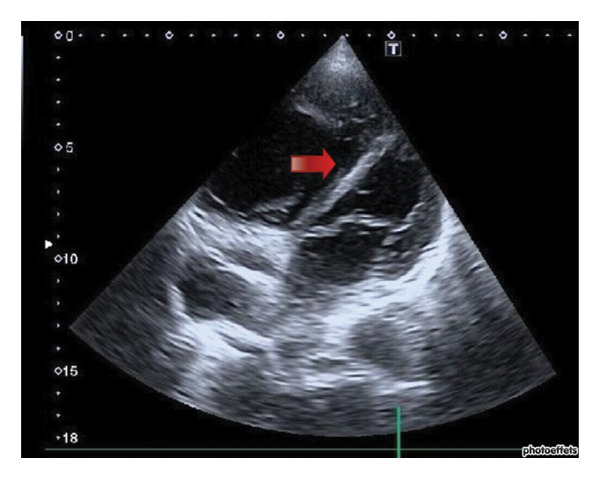
Apical four‐chamber view showing severe right ventricular dilation and septal flattening (“D‐sign”). The digital arrow indicates the flattened interventricular septum.

**FIGURE 2:**
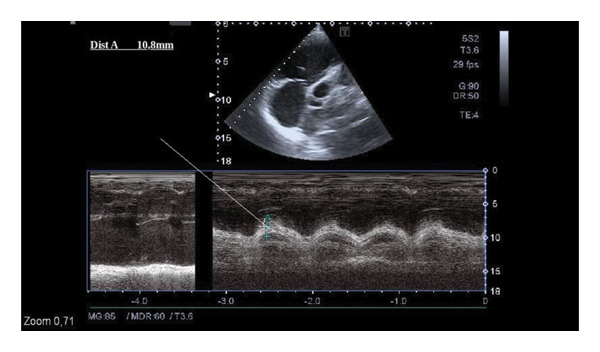
Measurement of tricuspid annular plane systolic excursion (TAPSE), demonstrating severely reduced longitudinal RV systolic function at 10 mm. The arrow shows the measurement cursor positions.

**FIGURE 3:**
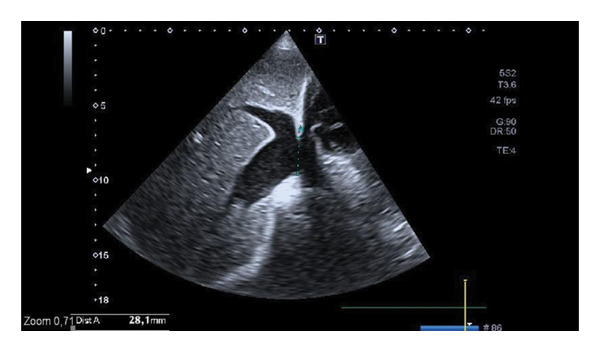
Subcostal view of the inferior vena cava (IVC) showing a plethoric, noncollapsing IVC measuring 28 mm in diameter. The arrow highlights the maximal diameter.

A 68‐year‐old patient underwent emergency laparotomy for sigmoid perforation with feculent peritonitis. Initial management of septic shock required 10 L of crystalloid over 24 h. By this point, lactate had risen to 4.5 mmol/L (normal < 2.0 mmol/L), and the patient had become anuric (< 50 mL/24 h). Bedside transthoracic echocardiography (TTE) showed severe RV dilation with interventricular septal flattening (D‐sign, Figure 1). TAPSE was 10 mm (Figure 2), indicating severe RV systolic dysfunction. The IVC, previously around 10 mm and collapsible, had become plethoric at 28 mm with absent respiratory variation (Figure 3). De‐resuscitation was initiated with intravenous furosemide 40 mg four times daily, while norepinephrine was titrated to maintain a mean arterial pressure ≥ 65 mmHg. Over the following 72 h, a cumulative negative fluid balance of approximately 6 L was achieved, accompanied by partial improvement in urine output, lactate levels, and RV size on repeat echocardiography. One week later, the patient developed recurrent sepsis from an anastomotic leak and died. The terminal deterioration was driven by this new septic insult rather than by de‐resuscitation itself.

## 3. Discussion

The transition from fluid responsiveness to fluid overload is easy to miss. Early resuscitation was necessary in this patient, but beyond a certain point further fluid worsened rather than improved the situation. This shift has been well described in septic shock and emphasizes why ongoing hemodynamic reassessment matters [4].

RV failure in sepsis has several overlapping mechanisms. Increased preload from fluid loading stretches an already stressed ventricle. Elevated pulmonary vascular resistance (PVR) raises afterload. Sepsis‐induced myocardial depression reduces contractile reserve, and because the two ventricles share the interventricular septum, severe RV dilation eventually impairs left ventricular filling as well [5, 6].

Lactic acidosis was an additional factor here. A lactate of 4.5 mmol/L indicates significant tissue hypoperfusion, and acidemia raises PVR through pulmonary vasoconstriction and direct vascular smooth muscle effects. It also depresses myocardial contractility by impairing calcium handling. These effects compound the hemodynamic consequences of fluid overload and are a reason to pursue lactate clearance in parallel with volume management.

Echocardiography is the most accessible tool for detecting RV failure at the bedside. RV dilation, septal flattening, reduced TAPSE, and IVC plethora are the main markers to look for [3, 7]. In critically ill patients, TAPSE below 16 mm has been linked to worse outcomes [8].

Venous congestion indices can add further information. Central venous pressure, hepatic vein Doppler, portal vein pulsatility, and renal venous flow patterns can each reflect the severity of fluid‐induced venous hypertension [7]. These parameters were not formally assessed in this patient—the IVC measurement was the primary tool used—but a multiparameter approach, when feasible, gives a more complete picture than IVC diameter alone.

Recent critical care literature increasingly supports that septic shock management should move away from fixed resuscitation protocols toward a more dynamic, individualized approach, with regular reassessment to determine when to stop fluids and when to start removing them [9, 10].

It is worth noting that this patient did not die of RV failure. He died of recurrent intraabdominal sepsis, which carries its own very high mortality [11]. De‐resuscitation worked temporarily, but could not offset the second septic insult.

## 4. Conclusion

This case shows how a fully developed RV failure syndrome—dilation, D‐sign, TAPSE of 10 mm, and plethoric IVC—can evolve silently during large‐volume resuscitation. Echocardiography identified it, but only after hemodynamic collapse had already occurred. Serial echocardiographic assessment from an earlier point in resuscitation could have detected the problem sooner and potentially allowed earlier de‐resuscitation. Repeated echocardiographic reassessment should be strongly considered in any patient with septic shock receiving high volumes of fluid.

## Author Contributions

Mohamed Achref Boubaker managed the patient, collected the clinical data, performed the echocardiographic assessment, wrote the manuscript, and approved the final version.

## Funding

No funding was received for the preparation of this case report.

## Ethics Statement

The author has nothing to report. This single‐patient case report used fully anonymized clinical data and did not require formal ethics committee approval according to local regulations.

## Consent

Written informed consent for publication of this case and accompanying images was obtained from the patient’s next of kin. All identifying information has been removed to ensure anonymity.

## Conflicts of Interest

The author declares no conflicts of interest.

## Data Availability

Data sharing is not applicable to this article as no datasets were generated or analyzed during the current study.
